# Deep learning algorithms to detect diabetic kidney disease from retinal photographs in multiethnic populations with diabetes

**DOI:** 10.1093/jamia/ocad179

**Published:** 2023-09-02

**Authors:** Bjorn Kaijun Betzler, Evelyn Yi Lyn Chee, Feng He, Cynthia Ciwei Lim, Jinyi Ho, Haslina Hamzah, Ngiap Chuan Tan, Gerald Liew, Gareth J McKay, Ruth E Hogg, Ian S Young, Ching-Yu Cheng, Su Chi Lim, Aaron Y Lee, Tien Yin Wong, Mong Li Lee, Wynne Hsu, Gavin Siew Wei Tan, Charumathi Sabanayagam

**Affiliations:** Yong Loo Lin School of Medicine, National University of Singapore, 117597, Singapore; School of Computing, National University of Singapore, 117417, Singapore; Singapore Eye Research Institute, Singapore National Eye Centre, 168751, Singapore; Department of Renal Medicine, Singapore General Hospital, 168753, Singapore; Singapore Eye Research Institute, Singapore National Eye Centre, 168751, Singapore; Singapore Eye Research Institute, Singapore National Eye Centre, 168751, Singapore; SingHealth Polyclinics, Singapore Health Services, 168582, Singapore; Westmead Institute for Medical Research, University of Sydney, NSW 2145, Australia; Centre for Public Health, Queen’s University Belfast, Belfast BT12 6BA, United Kingdom; Centre for Public Health, Queen’s University Belfast, Belfast BT12 6BA, United Kingdom; Centre for Public Health, Queen’s University Belfast, Belfast BT12 6BA, United Kingdom; Singapore Eye Research Institute, Singapore National Eye Centre, 168751, Singapore; Ophthalmology and Visual Science Academic Clinical Program, Duke-NUS Medical School, 169857, Singapore; Khoo Teck Puat Hospital, 768828, Singapore; Lee Kong Chian School of Medicine, Nanyang Technological University, Singapore, 308232, Singapore; Saw Swee Hock School of Public Health, National University of Singapore, 117549, Singapore; Department of Ophthalmology, University of Washington, Seattle, WA 98104, United States; Singapore Eye Research Institute, Singapore National Eye Centre, 168751, Singapore; Ophthalmology and Visual Science Academic Clinical Program, Duke-NUS Medical School, 169857, Singapore; School of Computing, National University of Singapore, 117417, Singapore; School of Computing, National University of Singapore, 117417, Singapore; Singapore Eye Research Institute, Singapore National Eye Centre, 168751, Singapore; Ophthalmology and Visual Science Academic Clinical Program, Duke-NUS Medical School, 169857, Singapore; Singapore Eye Research Institute, Singapore National Eye Centre, 168751, Singapore; Ophthalmology and Visual Science Academic Clinical Program, Duke-NUS Medical School, 169857, Singapore

**Keywords:** machine learning, diabetes, artificial intelligence, retina, screening, renal insufficiency

## Abstract

**Objective:**

To develop a deep learning algorithm (DLA) to detect diabetic kideny disease (DKD) from retinal photographs of patients with diabetes, and evaluate performance in multiethnic populations.

**Materials and methods:**

We trained 3 models: (1) image-only; (2) risk factor (RF)-only multivariable logistic regression (LR) model adjusted for age, sex, ethnicity, diabetes duration, HbA1c, systolic blood pressure; (3) hybrid multivariable LR model combining RF data and standardized z-scores from image-only model. Data from Singapore Integrated Diabetic Retinopathy Program (SiDRP) were used to develop (6066 participants with diabetes, primary-care-based) and internally validate (5-fold cross-validation) the models. External testing on 2 independent datasets: (1) Singapore Epidemiology of Eye Diseases (SEED) study (1885 participants with diabetes, population-based); (2) Singapore Macroangiopathy and Microvascular Reactivity in Type 2 Diabetes (SMART2D) (439 participants with diabetes, cross-sectional) in Singapore. Supplementary external testing on 2 Caucasian cohorts: (3) Australian Eye and Heart Study (AHES) (460 participants with diabetes, cross-sectional) and (4) Northern Ireland Cohort for the Longitudinal Study of Ageing (NICOLA) (265 participants with diabetes, cross-sectional).

**Results:**

In SiDRP validation, area under the curve (AUC) was 0.826(95% CI 0.818-0.833) for image-only, 0.847(0.840-0.854) for RF-only, and 0.866(0.859-0.872) for hybrid. Estimates with SEED were 0.764(0.743-0.785) for image-only, 0.802(0.783-0.822) for RF-only, and 0.828(0.810-0.846) for hybrid. In SMART2D, AUC was 0.726(0.686-0.765) for image-only, 0.701(0.660-0.741) in RF-only, 0.761(0.724-0.797) for hybrid.

**Discussion and conclusion:**

There is potential for DLA using retinal images as a screening adjunct for DKD among individuals with diabetes. This can value-add to existing DLA systems which diagnose diabetic retinopathy from retinal images, facilitating primary screening for DKD.

## Introduction

Diabetic kidney disease (DKD) is the leading cause of end-stage renal disease worldwide.^1^ Early detection of DKD would allow prompt preventive actions to limit excess morbidity and mortality in patients with diabetes mellitus (DM).^2^ Current guidelines recommend yearly blood tests to calculate estimated glomerular filtration rate (eGFR) from serum creatinine, or spot urine sampling for urine albumin/creatinine ratio (UACR).^3^^,^^4^ In individuals with DM, more frequent monitoring may be appropriate because of increased risk of progressive kidney disease relative to individuals with no diabetes.^3^^,^^4^ Furthermore, population screening for chronic kidney disease (CKD) among the DM subgroup was found to have acceptable cost effectiveness.^5^ However, studies in the United States,^6^ Australia,^7^ and Asia^8^ have reported suboptimal utilization or poor adherence to screening, and underdiagnosis of DKD. This remains a crucial barrier to early intervention.

Retinal photography is noninvasive and convenient, commonly used in primary care settings for screening of eye pathologies, particularly diabetic retinopathy. Because the retina and other end organs, including the kidneys, share similar structural, physiological (renin-angiotensin-aldosterone system), and pathogenic (inflammation, oxidative stress, endothelial dysfunction, microangiopathy) properties, the retinal vessels are an indirect representation of renal microvasculature.^9^ Clinically appreciable retinal microvascular changes have been associated with DKD,^10^^,^^11^ suggesting that retinal images contain substantial representative information of the kidney’s function. As the prevalence of diabetes grows worldwide,^12^ a noninvasive tool to screen for DKD would complement existing deep learning algorithm (DLA) systems to diagnose diabetic retinopathy from retinal images,^13^ facilitating primary care screening for complications of DM. Our group has previously developed and validated a DLA to detect CKD from retinal photographs in the general population in Singapore (RetiKid), which showed good performance in external datasets from Singapore and China.^14^ In this study, we developed and validated a DLA for detecting DKD (RetiKid-Diab) using retinal images from a clinic-based diabetic population. This model was compared with 2 other models—one using clinical risk factor (RF) data and another hybrid model combining a retinal imaging score and RF data—to assess if this could lead to improved DKD predictions compared to an image-only model.

## Methods

This study was approved by the Centralized Institutional Review Board (CIRB) of SingHealth, Singapore and conducted in accordance with the Declaration of Helsinki. Patients’ informed consent was exempted by the CIRB for the use of deidentified health information and retinal images. We performed a conventional development, validation, and external testing study on 3 models (retinal images only; RF only; hybrid) using retinal images and clinical data collected from 3 population-based studies. We developed and internally validated the models using data from the Singapore Integrated Diabetic Retinopathy Program (SiDRP).^15^ External testing was performed on 2 independent datasets: (1) Singapore Epidemiology of Eye Diseases (SEED) study^16^ and (2) Singapore Macroangiopathy and Microvascular Reactivity in Type 2 Diabetes (SMART2D) in Singapore.

### Definition of DKD

eGFR was calculated from serum creatinine using the CKD Epidemiology Collaboration (CKD-EPI) creatinine equation.^17^ Since SiDRP provided annual screening for retinopathy among individuals with diabetes since 2010, presence and absence of DKD were assessed in all visits where serum creatinine data were available. We included individuals with 4 or more screening visits. We defined DKD (cases) as those with eGFR < 60 mL/min/1.73 m^2^ on ≥2 consecutive visits between 3 months to 2 years apart (in the SiDRP development cohort; in external test cohorts, DKD was defined by a single visit). We defined no DKD (controls) as eGFR of ≥60 mL/min/1.73 m^2^ in all visits. Definitions of DKD in the external validation datasets (SEED and SMART2D) were the same as SiDRP.

### Training dataset for the DLA

**Figure 1.**
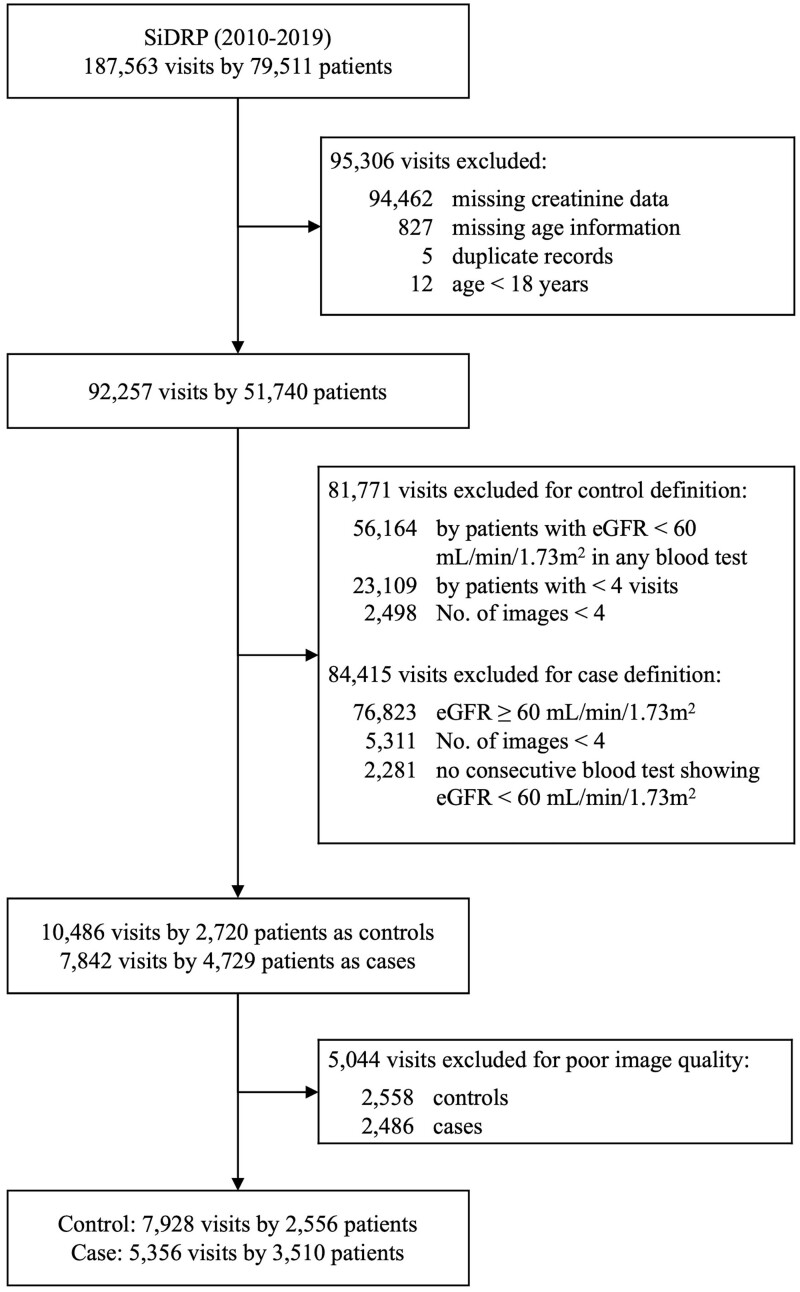
Flowchart showing detailing inclusion and exclusion of participants and images from SiDRP.

For development, data and retinal images were obtained from patients with DM who participated in SiDRP (2010-2019), a national-level, telemedicine-based program established in 2010 to optimize eye screening for a general urban diabetes population.^15^ For each patient, 2 retinal photographs (optic disc-centered and macula-centered) were taken from each eye after pupil dilation according to the Early Treatment for Diabetic Retinopathy Study (ETDRS) protocol using a digital retinal camera (TRC-NW200, Topcon, Japan). Figure 1 is a flowchart detailing the breakdown of participants and images included from SiDRP. 187 563 visits from 79 511 unique individuals were recruited and assessed for eligibility. After excluding samples missing creatinine or age data for eGFR calculation, having unstable CKD status, or poor image quality, 5356 cases (DKD positive visits) and 7928 controls (DKD negative visits) from SiDRP (total 6066 unique participants) were used for training and validation of the algorithm.

### External testing datasets for the DLA

Two datasets were used for external testing: SEED and SMART2D. SEED is an ongoing population-based study of Chinese, Malay, and Indian participants aged ≥40 years at baseline. Detailed methods of SEED have been published.^18^^,^^19^ After excluding those missing eGFR data or with poor quality images, data, and images from 1885 participants in SEED (798 cases, 1171 controls) were used for external testing, providing a total of 3938 fundus photographs (Table 1). SMART2D was a cross-sectional study conducted between 2011 and 2014 including 2057 adults aged 21-90 years with Type 2 DM. Detailed methods of SMART2D have been published.^20^ 1163 participants of SMART2D with eye screening visits were recruited for this study, of which 439 participants (227 cases, 485 controls) were eventually included, totaling 1424 fundus photographs.

**Table 1.** Baseline characteristics of participants.

	SiDRP (2010-2019)	SEED Diabetes cohort		SMART2D (2011-2014)	
	*N* = 13 284	*N* = 1969	*P*-value	*N* = 712	*P*-value
Number of unique participants	6066	1885		439	
Number of images	26 568	3938		1424	
Case: control	5356: 7928	798: 1171	.879	227: 485	<.001
Chronic kidney disease			<.001		<.001
Stage 3	4741 (88.5)	694 (87.0)		124 (54.6)	
Stage 4	558 (10.4)	79 (9.9)		44 (19.4)	
Stage 5	57 (1.1)	25 (3.1)		59 (26.0)	
Age (years)	64.1 (10.8)	64.0 (9.2)	.914	57.4 (10.7)	<.001
Sex			.060		.208
Female	6526 (49.1)	922 (46.8)		332 (46.6)	
Male	6758 (50.9)	1047 (53.2)		380 (53.4)	
Ethnicity			<.001		<.001
Malay	1833 (14.3)	705 (35.8)		159 (22.8)	
Indian	1145 (8.9)	868 (44.1)		165 (23.6)	
Chinese	9821 (76.7)	396 (20.1)		374 (53.6)	
Duration of Diabetes (years)	7.0 [3.0, 12.0]	7.0 [1.2, 14.7]	.306	13.0 [7.0, 20.0]	<.001
Hemoglobin A1C (%)	7.2 (1.2)	7.6 (1.6)	<.001	8.3 (1.8)	<.001
eGFR (mL/min/1.73 m^2^)	74.1 (27.1)	71.7 (25.8)	<.001	74.8 (33.6)	.504
Systolic blood pressure (mmHg)	130.2 (15.9)	143.8 (21.3)	<.001	143.5 (19.8)	<.001

Abbreviations: eGFR, estimated glomerular filtration rate; SiDRP, Singapore Integrated Diabetic Retinopathy Program; SEED, Singapore Epidemiology of Eye Diseases; SMART2D, Singapore Macroangiopathy and Microvascular Reactivity in Type 2 Diabetes data.

Data are *n* (%), mean (standard deviation, SD), or median [interquartile range, IQR]. Some numbers (eg, ethnicity) may not add up due to the presence of missing data. *P*-values were calculated between SiDRP and each external test set using Pearson’s chi-squared test, Student *t*-test, or Mann-Whitney *U*-test as appropriate for the variable.

### Risk factors

We used 6 classic RF (age, sex, ethnicity, duration of diabetes, HbA1c, and systolic blood pressure [SBP]) as predictors for the RF model. Age, sex, ethnicity, and duration of diabetes were self-reported in all cohorts. HbA1c and SBP were obtained from physical examination or laboratory tests in all datasets.

### Algorithm architecture and development

**Figure 2.**
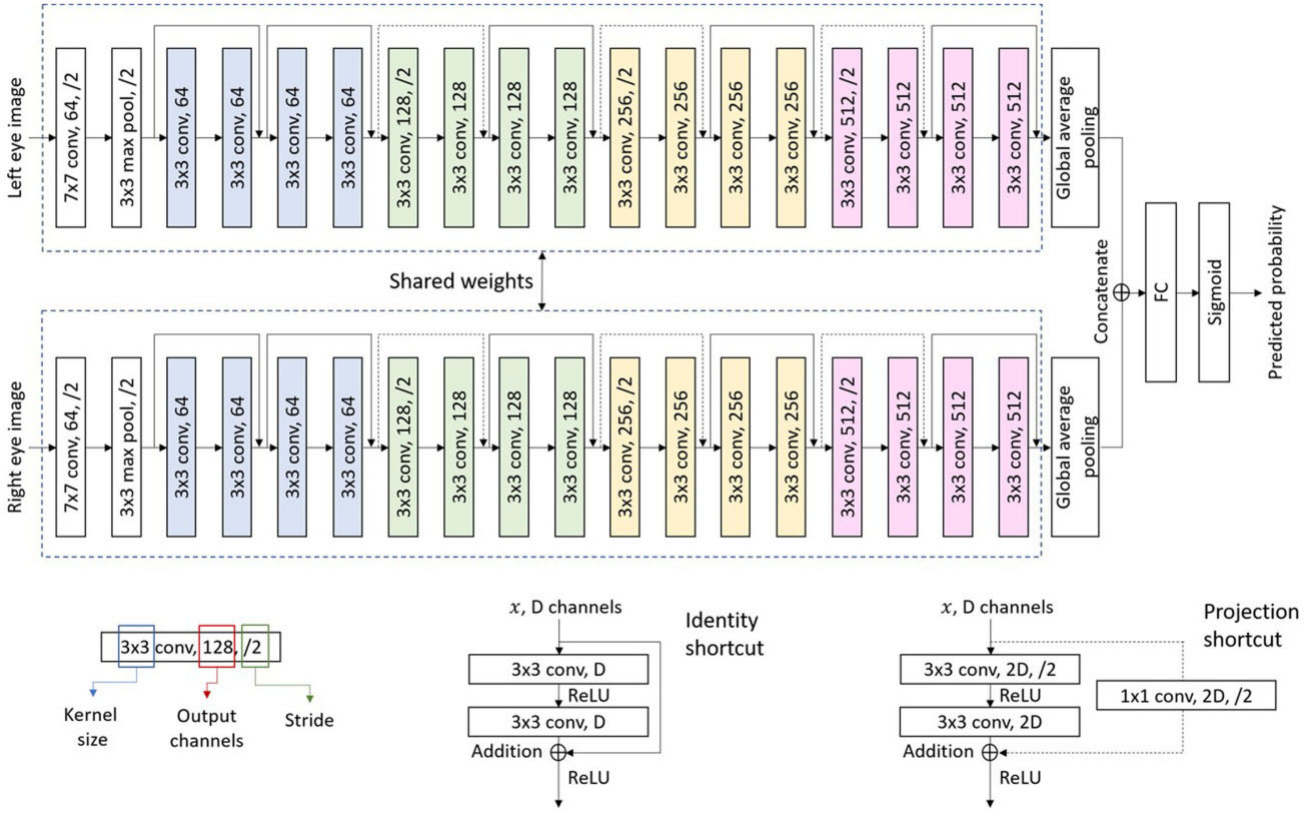
Convolutional neural network architecture for predicting diabetic kidney disease from retinal images.

The image-only DLA was trained on 26 568 retinal images from 6066 SiDRP participants. The DLA models were based off the ResNet18^21^ neural network architecture (Figure 2). The input layer takes 2 standardized macula-centered images (1 image per eye per participant) with resolution of 512 × 512. The output layer was a binary classifier with one node predicting the presence of DKD. During the training process, network parameters were initialized with weights pretrained using a large-scale diabetic retinopathy dataset (https://www.kaggle.com/competitions/diabetic-retinopathy-detection/data) to improve generalizability. For each image, the prediction given by the neural network is compared with its ground truth label, and parameters updated via backpropagation to reduce prediction error. We used 5-fold cross-validation to evaluate model performance, preserving the ratio of DKD cases and controls from the original dataset. The validation set had no overlap with the training set. The performance of the trained DLA was evaluated on the validation set by calculating the AUC combining the five sets of scores. Heatmaps were generated to identify the most important regions in a retinal image contributing to the DLA classification decision.

### Statistical analysis

We presented the characteristics of participants using number (%), mean (standard deviation, SD), or median [interquartile range, IQR] as appropriate for the variable. Model data from SiDRP were compared with each external validation set using Pearson’s chi-squared test, Fisher’s exact test, Student *t*-test, or Mann-Whitney *U*-test as appropriate (*P*-value <.05). We developed 3 models: (1) image-only model using retinal images; (2) RF-only model using multivariable logistic regression (LR) adjusted for age, sex, ethnicity, duration of diabetes, HbA1c, and SBP; (3) hybrid model using multivariable LR adjusted for age, sex, ethnicity, duration of diabetes, HbA1c, SBP, and standardized z-scores from the image-only model. Primary analysis was to evaluate the performance of the 3 models by calculating the area under the receiver operating characteristic curve (AUC), sensitivity, specificity, positive predictive value (PPV), and negative predictive value (NPV) at the optimal threshold defined by Youden’s J Index, on internal validation and external validation.

### Supplementary analysis

We performed several supplementary analyses including: (1) because misclassification of DKD would be common among those with eGFR near the normal range (55-60 mL/min/1.73 m^2^), we tested all 3 models using an alternate definition of DKD (eGFR <45 mL/min/1.73 m^2^; G3b and above). (2) We recalculated model performance when sensitivity or specificity were fixed at 80%. (3) We performed subgroup analysis calculating model accuracy for individuals in different CKD severity stages. (4) We externally validated the DLA in 2 predominantly Caucasian cohorts—a high-risk cohort for coronary artery disease (CAD) from the Australian Eye and Heart Study (AHES),^22^ and an older cohort from the Northern Ireland Cohort for the Longitudinal Study of Ageing (NICOLA).^23^ We performed these as supplementary analyses because the sample size and number of events were low. AHES is a cross-sectional study of 1680 participants (460 included in this study) presenting to the Westmead Hospital in Sydney for assessment of suspected CAD between 2009 and 2012.^22^ NICOLA is a cross-sectional study which collected health and lifestyle data from 8452 participants (265 included in this study) in Northern Ireland, aged ≥50 years. Baseline data were collected between 2013 and 2016. For AHES and NICOLA RF-only and Hybrid models, we did not adjust for ethnicity because a majority of AHES and NICOLA participants were Caucasian; in AHES, we also did not adjust for duration of diabetes as this information was unavailable. For missing values of duration of diabetes (in NICOLA), SBP and HbA1c, we used mean/mode imputation as per the SMART2D cohort. (5) Finally, we performed error analysis of false positive (FP) and false negative (FN) samples by key characteristics including albuminuria and presence of ocular diseases to gain some insights into the misclassification by the DLA.

## Results

Participant characteristics of each dataset are shown in Table 1. Characteristics of participants in SiDRP and SEED were similar, except for higher mean SBP in SEED (SiDRP: 130.2 [SD 15.9] mmHg vs SEED: 143.8 [SD 21.3] mmHg). There were several differences between the SMART2D dataset and the SiDRP or SEED datasets: (1) Participants from SMART2D were younger (57.4 years [SD 10.7]) than SiDRP (64.1 years [SD 10.8]) and SEED (64.0 years [SD 9.2]). (2) In terms of eGFR, CKD cases were generally more severe in SMART2D (tertiary care patients) than SiDRP (primary care patients) and SEED (population-based). There was a higher proportion of stage G4 and G5 CKD patients in SMART2D (G4: 19.4%; G5: 26.0%) than SiDRP (G4: 10.4%; G5: 1.1%) and SEED (G4: 9.9%; G5: 3.1%). (3) Ratio of cases (DKD positive visits) to controls (DKD negative visits) were lower in SMART2D (1 case: 2.1 controls) than SiDRP (1 case: 1.5 controls) and SEED (1 case: 1.5 controls). (4) Participants in SMART2D had a longer duration of diabetes (Median 13.0 years [IQR 7.0, 20.0]) than SiDRP (7.0 years [IQR 3.0, 12.0]) and SEED (7.0 years [IQR 1.2, 14.7]). (5) HbA1c was higher in participants from SMART2D (8.3% [SD 1.8]) than SiDRP (7.2% [SD 1.2]) and SEED (7.6% [SD 1.6]).

**Figure 3.**
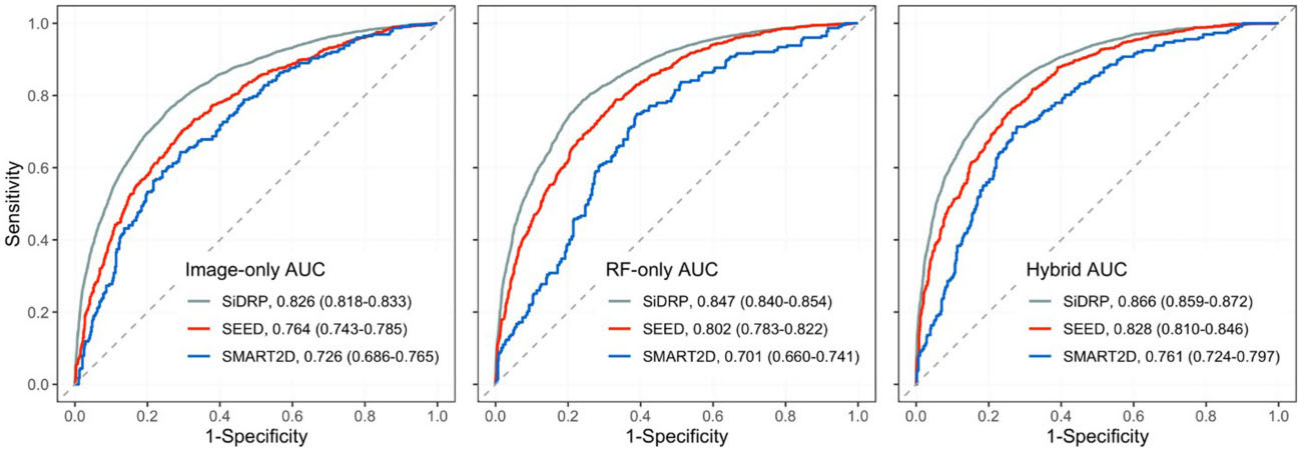
ROC curves for prediction of diabetic kidney disease in image-only, RF-only, and hybrid models.

Figure 3 shows the receiver operating characteristic (ROC) curve plots for the image-only DLA, RF-only multivariable LR model, and hybrid model. In SiDRP validation, AUC was 0.826 (95% CI 0.818-0.833) for image-only, 0.847 (0.840-0.854) for RF-only, and 0.866 (0.859-0.872) for hybrid. In external validation with SEED, AUC was 0.764 (0.743-0.785) for image-only, improving to 0.802 (0.783-0.822) for RF-only and 0.828 (0.810-0.846) for hybrid. In SMART2D external validation, AUC was 0.726 (0.686-0.765) for image-only, decreasing to 0.701 (0.660-0.741) in RF-only and improving to 0.761 (0.724-0.797) for hybrid. Table 2 provides additional performance metrics to compare both models in internal validation and external test sets, at the optimal cut-off point defined by Youden’s J Index. In SiDRP, the image-only model had a sensitivity of 76% and specificity of 75%. The RF-only and hybrid models had sensitivity ranging from 78% to 79% and specificity ranging from 76% to 77%. In SEED, the image-only model had a sensitivity of 70% and specificity of 71%. The RF-only and hybrid models had higher sensitivities at 79% and 76%, respectively, while specificity was 67% and 74%, respectively. In SMART2D, image-only model had a sensitivity of 64% and specificity of 71%. RF-only and hybrid models had higher sensitivities at 75% and 71%, respectively, while specificity was 61% and 72%, respectively. NPV for the image-only model was 82% in SiDRP, 78% in SEED, and 81% in SMART2D. In RF-only and hybrid models, NPV levels were generally higher, ranging from 82% to 86% in all datasets. PPV values were consistently lower than NPV in all models and all datasets.

**Table 2.** Performance of the 3 models in internal and external test sets at the optimal thresholds by Youden’s J Index.

	AUC (95% CI)	Sensitivity	Specificity	PPV	NPV
SiDRP, *n* = 13 284					
Image-only	0.826 (0.818-0.833)	0.76	0.75	0.67	0.82
RF-only	0.847 (0.840-0.854)	0.78	0.76	0.68	0.85
Hybrid	0.866 (0.859-0.872)	0.79	0.77	0.69	0.86
SEED, *n* = 1969					
Image-only	0.764 (0.743-0.785)	0.70	0.71	0.62	0.78
RF-only	0.802 (0.783-0.822)	0.79	0.67	0.61	0.82
Hybrid	0.828 (0.810-0.846)	0.76	0.74	0.66	0.82
SMART2D, *n* = 712					
Image-only	0.726 (0.686-0.765)	0.64	0.71	0.51	0.81
RF-only	0.701 (0.660-0.741)	0.75	0.61	0.47	0.84
Hybrid	0.761 (0.724-0.797)	0.71	0.72	0.55	0.84

Abbreviations: AUC, area under the receiver operating characteristics curve; SiDRP, Singapore Integrated Diabetic Retinopathy Program; SEED, Singapore Epidemiology of Eye Diseases; SMART2D, Singapore Macroangiopathy and Microvascular Reactivity in Type 2 Diabetes data.

RF-only: Logistic regression adjusted for age, sex, ethnicity, diabetes duration, HbA1c, and systolic blood pressure. Hybrid: Logistic regression adjusted for age, sex, ethnicity, diabetes duration, HbA1c, systolic blood pressure, and the Image-only predicted z scores.

Table 3 shows the results of sensitivity analysis using the alternate definition of DKD (eGFR <45 mL/min/1.73 m^2^). Model performance improved for all models in SiDRP and SEED, and the image-only model in SMART2D. In the image-only model, AUC improved from 0.826 (0.818-0.833) to 0.851 (0.843, 0.860) in SiDRP; from 0.764 (0.743-0.785) to 0.785 (0.757-0.813) in SEED, and from 0.726 (0.686-0.765) to 0.759 (0.718-0.800) in SMART2D. In hybrid models, AUCs improved from 0.866 (0.859-0.872) to 0.887 (0.880-0.895) in SiDRP; from 0.828 (0.810-0.846) to 0.851 (0.829-0.874) in SEED; and from 0.761 (0.724-0.797) to 0.765 (0.723-0.806) in SMART2D. The highest AUC in external validation was achieved in SEED hybrid model, with an AUC of 0.851 (0.829-0.874).

**Table 3.** AUC (95% CI) of the 3 models excluding DKD Stage G3a (eGFR 45-60 mL/min/1.73 m^2^).

	Case: control	Image-only (95% CI)	RF-only (95% CI)	Hybrid (95% CI)
SiDRP, *n* = 10 311	2383: 7928	0.851 (0.843-0.860)	0.868 (0.860-0.877)	0.887 (0.880-0.895)
SEED, *n* = 1508	337: 1171	0.785 (0.757-0.813)	0.822 (0.797-0.848)	0.851 (0.829-0.874)
SMART2D, *n* = 645	160: 485	0.759 (0.718-0.800)	0.678 (0.631-0.725)	0.765 (0.623-0.806)

Abbreviations: AUC, area under the receiver operating characteristics curve; SiDRP, Singapore Integrated Diabetic Retinopathy Program; SEED, Singapore Epidemiology of Eye Diseases; SMART2D, Singapore Macroangiopathy and Microvascular Reactivity in Type 2 Diabetes data.

Optimal probability threshold chosen by Youden’s J Index.

We also calculated model performances when sensitivity or specificity were fixed at 80% (Table S1). When sensitivity was fixed at 80%; the image-only model had a specificity of 70% in SiDRP, 57% in SEED, and 51% in SMART2D. Specificities of the RF-only and hybrid models ranged from 51% to 77%. When specificity was fixed at 80%; the image-only model had a sensitivity of 70% in SiDRP, 58% in SEED, and 53% in SMART2D. Sensitivities of the RF-only and hybrid models ranged from 40% to 77%. Next, we performed subgroup analysis for individuals in different eGFR categories. When fixed at 80% sensitivity, the image-only model was able to detect 81%-82% of the cases in Stage G3B CKD, 87%-93% of the cases in Stage G4 CKD, and 82%-96% of the Stage G5 DKD.

In our error analysis of the image-only model, images from controls (non-DKD) are more likely to be labeled positive (FP) if they were taken from patients that were older (66.2 [7.3] vs 56.2 [8.1] years), of Chinese ethnicity, had longer duration of diabetes (6.0 [3.0, 9.0] vs 4.0 [2.0, 8.0] years), higher SBP (129.9 [15.8] vs 126.0 [14.4] mmHg), lower HbA1c (7.0 [1.0] vs 7.2 [1.2] %), or had cataracts (*n* = 28/1131, 2.5% vs *n* = 9/4007, 0.2%). Images from DKD cases were more likely to be labeled negative (FN) if they were taken from patients that were younger (65.1 [7.7] vs 75.1 [7.5] years), of Malay ethnicity, had lower SBP (128.0 [16.9] vs 131.7 [17.1] mmHg), or did not have cataracts (*n* = 1/535, 0.2% vs *n* = 68/985, 6.9%).

Finally, in our supplementary analysis on the AHES and NICOLA cohort, characteristics of AHES and NICOLA participants are provided in Table S2. Regarding model performance in AHES, the image-only model achieved an AUC of 0.670 (0.612-0.729), which was slightly lower than its performance in the 3 main datasets. Otherwise, the RF-only and hybrid models performed similarly, achieving AUCs of 0.685 (0.626-0.745) and 0.695 (0.640-0.751) respectively (Figure S1). Regarding model performance in NICOLA, the image-only model achieved an AUC of 0.638 (0.562-0.714), which was lower than its performance in the 3 main datasets. However, the RF-only and hybrid models performed well, reaching AUCs of 0.721 (0.652-0.790) and 0.710 (0.640-0.779) respectively (Figure S1).

## Discussion

We developed and validated a DLA for detecting DKD from retinal images (RetiKid-Diab), aiming to determine if the models are sufficiently robust to screen individuals with diabetes in the primary care setting. To our knowledge, this is the first study that attempts to predict DKD from retinal images in a population with diabetes, augmenting existing reports of CKD diagnosis using fundus images in the general population.^14^^,^^24^ Our models showed reasonable performance, faring well in internal validation (AUC image-only = 0.826 [0.818-0.833]), with moderate performance in external validation (AUC image-only = 0.764 [0.743-0.785] in SEED; 0.726 [0.686-0.765] in SMART2D). In particular, the image-only model performed comparably well in all datasets compared to the RF-only model. That being said, the hybrid models, comprising both retinal image and RF, performed better than the image-only or RF-only versions across all datasets. Taken together, these results suggest that there is potential for DLA using retinal images as a screening adjunct for DKD among individuals with diabetes, alongside standard screening methods. Inclusion of common, readily acquirable RFs will add some value to the performance of the algorithm, if it were translated into the primary care setting.

An important consideration for any artificial intelligence (AI)-based screening system is its clinical relevance. DKD has a long asymptomatic phase, and early detection is critical for optimal management. It is widely reflected in medical guidelines that people with diabetes should be regularly evaluated for DKD, typically with an annual urine test for albuminuria and a blood test for serum creatinine to estimate GFR.^25^ However, current screening rates are suboptimal^26^; in a systematic review exploring screening rates among individuals with diabetes for diabetes-related complications, de Jong et al^27^ reported that two-thirds of studies described nephropathy screening rates of less than 70%. In the Korean National Health and Nutrition Examination Survey, only 40.5% of patients with diabetes received screening for diabetic nephropathy during the previous year, even though they knew that they had diabetes.^28^ While an AI-based retinal image screening system for DKD may not replace current screening methods in the near future, it has potential to serve as a screening adjunct, to improve worldwide screening rates. Firstly, telemedicine for diabetic retinopathy screening among those with diabetes has remained strongly cost-effective compared with in-person office screening.^29^ Secondly, AI-based diabetic retinopathy screening programs have begun real-world implementation^30^; a noninvasive, low cost, point-of-care DKD screening tool that uses the same input (retinal fundus photographs) provides the opportunity for simultaneous screening of 2 major microvascular complications of diabetes (DR+DKD) at the population level. For example, in Singapore, patients on follow-up for diabetes are routinely screened for referable DR using a nationwide implemented deep learning software—Singapore Eye Lesions Analyzer (SELENA).^31^ Using the same retinal images, patients could be screened for DKD as well.

Our results had several notable trends. There was a reduction in performance of the image-only model on SMART2D external validation, with particularly poorer specificity and PPV. However, this was also noticed with the RF-only and Hybrid models. The proportion of DKD cases in SMART2D (31.9%) was lower than SiDRP (40.3%) or SEED (40.5%), which could explain the lower PPV. Comparing this to real-world rates, reported prevalence of DKD among patients with diabetes vary greatly from approximately 20%-40%,^32^^,^^33^ depending on the study population and presence of cardiovascular comorbidities. To increase PPV in populations with low prevalence of DKD, our DLA could be applied to higher risk individuals, including individuals with diabetes that are older, have poorer glycemic control, or multiple cardiovascular comorbidities. We also suggest applying this tool as part of a 2-stage screening, where a noninvasive DLA pegged at higher sensitivity can be applied first; individuals who screen positive are recalled for further testing with serum creatinine, to exclude false positives. There were 2 other notable trends regarding the image-only model in our results. First, performance of the image-only model improved in all datasets when the stricter definition of DKD (eGFR <45 mL/min/1.73 m^2^) was used. Second, the accuracy of the image-only model when sensitivity was fixed at 80% increased with the severity of DKD. This would be beneficial for a community screening adjunct, to safeguard that moderate/severe cases are less likely to be missed. Next, in our error analysis of the image-only model, FP labels occurred in patients that were older, had a longer duration of diabetes, or higher SBP. Conversely, FN labels occurred in patients that were younger and had lower SBP. This trend suggests that a positive label is associated with patients with a higher cardiovascular risk profile (and vice versa), which is not unexpected. FP labels were more likely in patients with lower HbA1c, while FN labels more likely in patients with higher HbA1c. It seems contradictory that better glycemic control is associated with a positive label. FP labels were more likely to occur in patients with cataracts (and vice versa)—this could be related to the known association between CKD and cataracts.^34^ The presence of cataracts can also affect the quality of fundus images, and in turn the performance of the DLA. Patients with albuminuria were more likely to have FP labels, suggesting that the DLA is identifying signals from those with early renal impairment (for example, stage 1 and 2 DKD which we classified as controls). Finally, on external validation, we observed slight reductions in AUCs for the image-only model. The small sample size of these external validation datasets (*n* = 460 in AHES, *n* = 265 in NICOLA) could explain the reduced, albeit modest, image-only model performance. With further training on more ethnically diverse datasets, our model has the potential to generalize well in populations of varying ethnic predominance, which is advantageous for any AI-based community screening device.

Several image-based DLAs have been created to screen for CKD,^14^^,^^24^^,^^35^^,^^36^ but not for DKD (Table 4). The RetiKid-Diab algorithm we describe in this article is a “sister” algorithm to RetiKid,^14^ developed by our group in 2020. In our previous report,^14^ RetiKid was also tested on a subgroup of individuals with diabetes (ie, predicting DKD), achieving better AUCs than RetiKid-Diab on internal validation (RetiKid hybrid AUC: 0.925; RetiKid-Diab hybrid AUC: 0.876). The algorithms used were inherently different in architecture—RetiKid is a neural network, able to utilize nonlinear associations and interaction terms. RetiKid-Diab image-only was also a neural network. However, RetiKid-Diab RF-only and hybrid models are multivariable LR models, which cannot utilize interaction or high-order terms for prediction. We note that a direct comparison of AUCs does not provide a full picture of model performance, and we suggest further evaluation of existing retina-based CKD screening tools in diabetic cohorts.

**Table 4.** Performance of deep-learning algorithms related to chronic kidney disease in current literature.

Study	Purpose	Limitations	Input	Model	Dataset	Validation	AUC	Sensitivity	Specificity
Betzler 2023^a^ Current Study	To detect DKD in a diabetic population. DKD defined as eGFR < 60 mL/min/1.73 m^2^ (category G3 and above) on ≥2 consecutive visits between 3 months to 2 years apart.	Reduced performance when validated on Caucasian datasets.	Retinal fundus images	CNN	31 930 images SiDRP SEED SMART2D	Internal	0.854 (Image) 0.847 (RF) 0.876 (Hybrid)	0.77 (Image) 0.78 (RF) 0.81 (Hybrid)	0.78 (Image) 0.76 (RF) 0.79 (Hybrid)
						External 1	0.744 (Image) 0.802 (RF) 0.821 (Hybrid)	0.80 (Image) 0.79 (RF) 0.74 (Hybrid)	0.59 (Image) 0.67 (RF) 0.76 (Hybrid)
						External 2	0.724 (Image) 0.685 (RF) 0.745 (Hybrid)	0.77 (Image) 0.78 (RF) 0.74 (Hybrid)	0.63 (Image) 0.56 (RF) 0.68 (Hybrid)
Sabanayagam 2020^14^	To detect CKD in a general population CKD defined as eGFR < 60 mL/min/1.73 m^2^ (category G3 and above) in either of 2 consecutive visits	No external validation on non-Asian datasets	Retinal fundus images	CNN	23 516 images SEED SP2 BES	Internal	0.911 (Image) 0.916 (RF) 0.938 (Hybrid)	0.83 (Image) 0.82 (RF) 0.84 (Hybrid)	0.83 (Image) 0.84 (RF) 0.85 (Hybrid)
						External 1	0.733 (Image) 0.829 (RF) 0.810 (Hybrid)	0.70 (Image) 0.73 (RF) 0.74 (Hybrid)	0.70 (Image) 0.80 (RF) 0.75 (Hybrid)
						External 2	0.835 (Image) 0.887 (RF) 0.858 (Hybrid)	0.75 (Image) 0.79 (RF) 0.79 (Hybrid)	0.75 (Image) 0.82 (RF) 0.79 (Hybrid)
						Internal, Diabetic Subgroup	0.889 (Image) 0.899 (RF) 0.925 (Hybrid)	—	—
Zhang 2021^24^	To detect CKD in a general population CKD defined as eGFR <60 mL/min/1.73 m^2^ or eGFR >60 mL/min/1.73 m^2^ with albuminuria, confirmed in ≥2 visits separated by 3 months	Training and external validation sets were of similar ethnicity (Chinese)	Retinal fundus images	CNN	115 344 images CC-FII Guangdong COACS	External 1	0.918 (Image) 0.861 (RF) 0.930 (Hybrid)	—	—
						External 2	0.885 (Image) 0.842 (RF) 0.898 (Hybrid)	—	—
Kang 2020^35^	To detect early renal function impairment in a general population Early renal function impairment defined as eGFR <90 mL/min/1.73 m^2^	No external validation	Retinal fundus images	CNN	25 706 images CGMH Taiwan	Internal	0.810 (Image)	0.830 (Image)	0.620 (Image)
Kuo 2019^36^	To predict kidney function from kidney ultrasound images CKD defined as eGFR < 60 mL/min/1.73 m^2^ (category G3 and above)	No external validation	Kidney ultrasound images	CNN	4505 images CMUH Taiwan	Internal	0.904 (Image)	0.921 (Image)	0.606 (Image)

Abbreviations: AUC, area under the receiver operating curve; BES, Beijing Eye Study; CC-FII, China Consortium of Fundus Image Investigation; CGMH, Chang Gung Memorial Hospital; CKD, chronic kidney disease; CMUH, China Medical University Hospital in Taiwan; CNN, convolutional neural network; COACS, China Suboptimal Health Cohort Study; DKD, diabetic kidney disease; eGFR, estimated glomerular filtration rate; RF, Risk Factors; SiDRP, Singapore Integrated Diabetic Retinopathy Program; SEED, Singapore Epidemiology of Eye Disease; SMART2D, Singapore Macroangiopathy and Microvascular Reactivity in Type 2 Diabetes; SP2, Singapore Prospective Study Program.

a The current study is the only one conducted for DKD in a diabetic population. All other published studies listed here were conducted in a general population.

**Figure 4.**
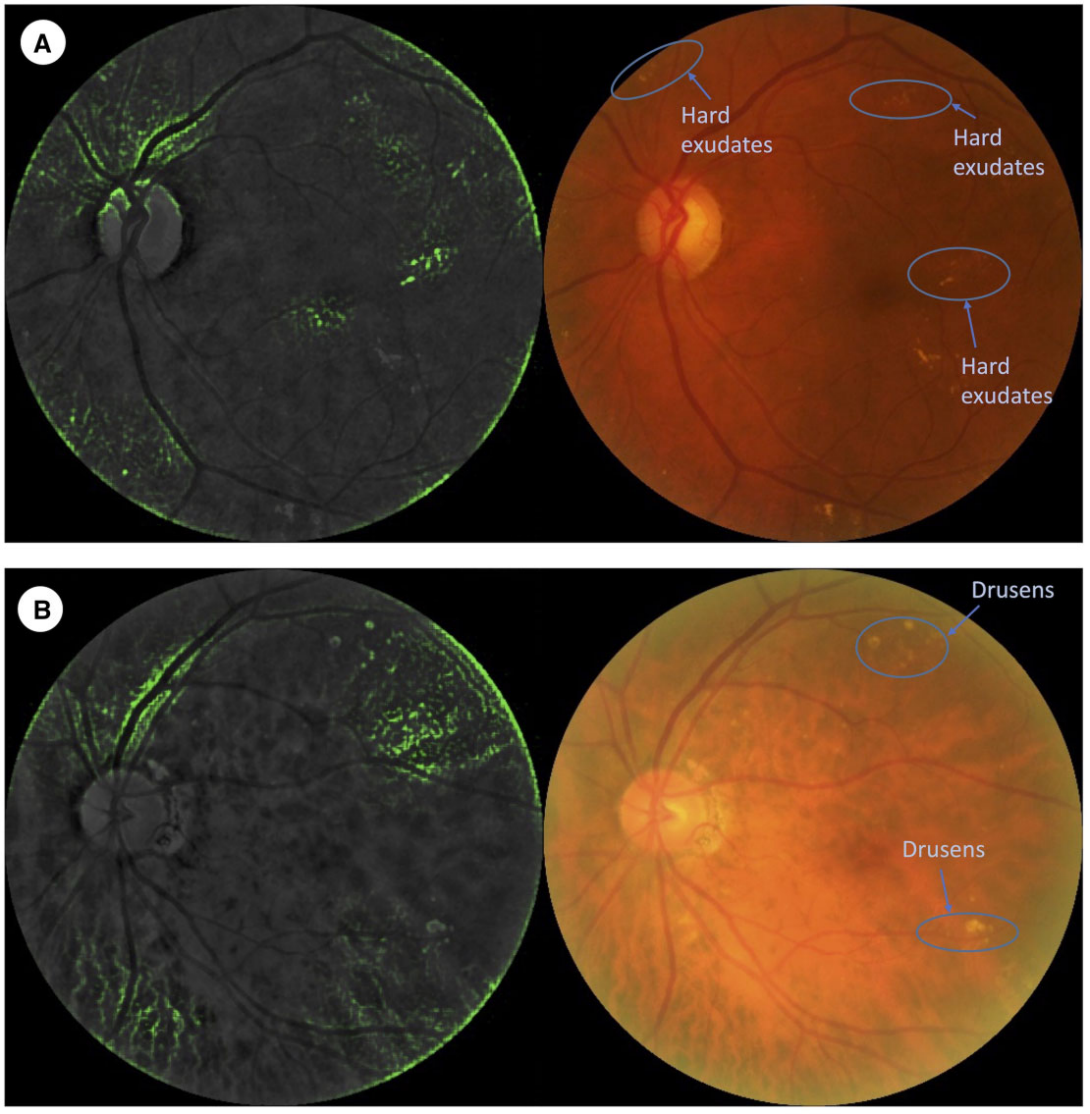
Heatmaps of diabetic kidney disease controls and cases.

The strengths of our study include the validation of our DLA on independent cohorts with similar imaging and DKD diagnostic protocols. Next, we had a robust ground truth, where DKD was defined based on at least 2 measurements of eGFR on consecutive visits, potentially reducing misclassification of DKD cases. In addition, we explored the utility of hybrid models with additional clinical RF data, which showed improvement relative to image-only models. Nevertheless, our study has several limitations. First, because our study was based in an Asian population (Chinese, Indian, and Malay), our DLA would be more relevant in Asian countries with a high burden of diabetes and DKD. Further evaluation of its generalizability in non-Asian populations, and in more diverse demographic cohorts, may improve clinical utility and diagnostic accuracy. We explored this with our supplementary analyses on the predominantly Caucasian AHES (Australia) and NICOLA (Northern Ireland) cohorts, which demonstrated modest performance. Second, we did not have data on albuminuria for all participants, thus we could not incorporate albuminuria levels into the prediction. Third, we had a low representation of Stage G5 cases in the SiDRP training set, while Stage G5 cases were over-represented in SMART2D. Fourth, while heatmaps (Figure 4) indicated microvascular changes characteristic of retinopathy, it is unclear what specific features were used by the DLA to identify DKD. This is a problem faced by most existing image-based DLAs. A multistep algorithm that detects characteristic microvascular changes and uses these features to predict DKD is possible, although this might overcomplicate the prediction process without a substantial increase in performance. Fifth, our current model underperforms relative to our prior work in a general population.^14^ However, this could be because the training dataset of our prior work was under a SEED research dataset which has less noisy labels. Our current model was trained on a real-world dataset which could account for the difference in performance. We attempted other machine learning classifiers (such as random forest and support vector machine), but overall the results were suboptimal to LR. We also attempted vision transformers (ViT) as the deep learning image model, performance was comparable, but it was computationally intensive and the training took longer.

In conclusion, our study shows the potential of a retina image DLA to screen for DKD among individuals with diabetes. Since access to digital retinal photography is increasing at the primary care level, a retinal image-based DLA, if adopted, has the potential to improve DKD screening rates worldwide, as an adjunct to existing laboratory methods. Next steps might include validating the algorithm in non-Asian populations, among young patients with diabetes (ie, T1DM patients) and performing implementation and cost-effectiveness studies.

## Supplementary Material

ocad179_Supplementary_Data

## Data Availability

As the study involves human participants, the data cannot be made freely available in the manuscript, the supplemental files, or a public repository due to ethical restrictions. Nevertheless, the data are available from the Singapore Eye Research Institutional Ethics Committee for researchers who meet the criteria for access to confidential data. Interested researchers can send data access requests to the Singapore Eye Research Institute using the following email address: seri@seri.com.sg.

## References

[1] Deng Y , Li N, Wu Y, et al Global, regional, and national burden of diabetes-related chronic kidney disease from 1990 to 2019. Front Endocrinol (Lausanne). 2021;12:672350. 10.3389/fendo.2021.672350 34276558 PMC8281340

[2] Thornton Snider J , Sullivan J, van Eijndhoven E, et al Lifetime benefits of early detection and treatment of diabetic kidney disease. PLoS One 2019;14(5):e0217487. 10.1371/journal.pone.0217487 31150444 PMC6544227

[3] Levin A , Stevens PE, Bilous RW, et al Kidney Disease: Improving Global Outcomes (KDIGO) CKD Work Group. KDIGO 2012 clinical practice guideline for the evaluation and management of chronic kidney disease. Kidney Int Suppl. 2013;3(1):1-150.

[4] American Diabetes Association. 11. Microvascular complications and foot care: standards of medical care in diabetes—2019. Diabetes Care 2019;42(Suppl_1):S124-S38. 30559237 10.2337/dc19-S011

[5] Manns B , Hemmelgarn B, Tonelli M, et al; Alberta Kidney Disease Network. Population based screening for chronic kidney disease: cost effectiveness study. BMJ 2010;341:c5869. 10.1136/bmj.c5869 21059726 PMC2975430

[6] Szczech LA , Stewart RC, Su HL, et al Primary care detection of chronic kidney disease in adults with type-2 diabetes: the ADD-CKD Study (awareness, detection and drug therapy in type 2 diabetes and chronic kidney disease). PLoS One 2014;9(11):e110535. 10.1371/journal.pone.0110535 25427285 PMC4245114

[7] Manski-Nankervis JE , Thuraisingam S, Lau P, et al Screening and diagnosis of chronic kidney disease in people with type 2 diabetes attending Australian general practice. Aust J Prim Health. 2018;24(3):280-286. 10.1071/py17156 29807557

[8] Luk AO , Li X, Zhang Y, et al; JADE Study Group. Quality of care in patients with diabetic kidney disease in Asia: the Joint Asia Diabetes Evaluation (JADE) Registry. Diabet Med. 2016;33(9):1230-1239. 10.1111/dme.13014 26511783

[9] Wong CW , Wong TY, Cheng CY, Sabanayagam C. Kidney and eye diseases: common risk factors, etiological mechanisms, and pathways. Kidney Int. 2014;85(6):1290-1302. 10.1038/ki.2013.491 24336029

[10] Xu X , Gao B, Ding W, et al Retinal image measurements and their association with chronic kidney disease in Chinese patients with type 2 diabetes: the NCD study. Acta Diabetol. 2021;58(3):363-370. 10.1007/s00592-020-01621-6 33098472

[11] Nusinovici S , Sabanayagam C, Lee KE, et al Retinal microvascular signs and risk of diabetic kidney disease in Asian and White populations. Sci Rep. 2021;11(1):4898. 10.1038/s41598-021-84464-7 33649427 PMC7921402

[12] Saeedi P , Petersohn I, Salpea P, et al; IDF Diabetes Atlas Committee. Global and regional diabetes prevalence estimates for 2019 and projections for 2030 and 2045: results from the International Diabetes Federation Diabetes Atlas, 9(th) edition. Diabetes Res Clin Pract. 2019;157:107843. 10.1016/j.diabres.2019.107843 31518657

[13] Gulshan V , Peng L, Coram M, et al Development and validation of a deep learning algorithm for detection of diabetic retinopathy in retinal fundus photographs. JAMA 2016;316(22):2402-2410. 10.1001/jama.2016.17216 27898976

[14] Sabanayagam C , Xu D, Ting DSW, et al A deep learning algorithm to detect chronic kidney disease from retinal photographs in community-based populations. Lancet Digit Health. 2020;2(6):e295-e302. 10.1016/s2589-7500(20)30063-7 33328123

[15] Nguyen HV , Tan GS, Tapp RJ, et al Cost-effectiveness of a National Telemedicine Diabetic Retinopathy Screening Program in Singapore. Ophthalmology 2016;123(12):2571-2580. 10.1016/j.ophtha.2016.08.021 27726962

[16] Majithia S , Tham YC, Chee ML, et al Cohort profile: the Singapore Epidemiology of Eye Diseases study (SEED). Int J Epidemiol. 2021;50(1):41-52. 10.1093/ije/dyaa238 33393587

[17] Inker LA , Schmid CH, Tighiouart H, et al; CKD-EPI Investigators. Estimating glomerular filtration rate from serum creatinine and cystatin C. N Engl J Med. 2012;367(1):20-29. 10.1056/NEJMoa1114248 22762315 PMC4398023

[18] Foong AW , Saw S-M, Loo J-L, et al Rationale and methodology for a population-based study of eye diseases in Malay people: the Singapore Malay Eye Study (SiMES). Ophthalmic Epidemiol. 2007;14(1):25-35. 17365815 10.1080/09286580600878844

[19] Lavanya R , Jeganathan VSE, Zheng Y, et al Methodology of the Singapore Indian Chinese Cohort (SICC) eye study: quantifying ethnic variations in the epidemiology of eye diseases in Asians. Ophthalmic Epidemiol. 2009;16(6):325-336. 19995197 10.3109/09286580903144738

[20] Zhang X , Liu JJ, Sum CF, et al Ethnic disparity in central arterial stiffness and its determinants among Asians with type 2 diabetes. Atherosclerosis 2015;242(1):22-28. 10.1016/j.atherosclerosis.2015.06.019 26162317

[21] He K , Zhang X, Ren S, Sun J. Deep residual learning for image recognition. In: *Proceedings of the IEEE Conference on Computer Vision and Pattern Recognition*; 2016; Las Vegas, NV.

[22] Gopinath B , Chiha J, Plant AJ, et al Associations between retinal microvascular structure and the severity and extent of coronary artery disease. Atherosclerosis 2014;236(1):25-30. 10.1016/j.atherosclerosis.2014.06.018 25010900

[23] O’Neill RA , Maxwell AP, Kee F, et al Association of retinal venular tortuosity with impaired renal function in the Northern Ireland Cohort for the Longitudinal Study of Ageing. BMC Nephrol. 2020;21(1):382. 10.1186/s12882-020-02031-0 32883218 PMC7469276

[24] Zhang K , Liu X, Xu J, et al Deep-learning models for the detection and incidence prediction of chronic kidney disease and type 2 diabetes from retinal fundus images. Nat Biomed Eng. 2021;5(6):533-545. 10.1038/s41551-021-00745-6 34131321

[25] McGrath K , Edi R. Diabetic kidney disease: diagnosis, treatment, and prevention. Am Fam Physician. 2019;99(12):751-759. 31194487

[26] Alicic RZ , Rooney MT, Tuttle KR. Diabetic kidney disease: challenges, progress, and possibilities. Clin J Am Soc Nephrol. 2017;12(12):2032-2045. 10.2215/cjn.11491116 28522654 PMC5718284

[27] de Jong M , Peters SAE, de Ritter R, et al Sex disparities in cardiovascular risk factor assessment and screening for diabetes-related complications in individuals with diabetes: a systematic review. Front Endocrinol (Lausanne). 2021;12:617902. 10.3389/fendo.2021.617902 33859615 PMC8043152

[28] Byun SH , Ma SH, Jun JK, Jung KW, Park B. Screening for diabetic retinopathy and nephropathy in patients with diabetes: a nationwide survey in Korea. PLoS One 2013;8(5):e62991. 10.1371/journal.pone.0062991 23667557 PMC3648467

[29] Siegel KR , Ali MK, Zhou X, et al Cost-effectiveness of interventions to manage diabetes: has the evidence changed since 2008? Diabetes Care 2020;43(7):1557-1592. 10.2337/dci20-0017 33534729

[30] Dong X , Du S, Zheng W, Cai C, Liu H, Zou J. Evaluation of an artificial intelligence system for the detection of diabetic retinopathy in Chinese community healthcare centers. Front Med (Lausanne). 2022;9:883462. 10.3389/fmed.2022.883462 35479949 PMC9035696

[31] Wong DCS , Kiew G, Jeon S, Ting D. Singapore Eye Lesions Analyzer (SELENA): the deep learning system for retinal diseases. In: Grzybowski A, ed. Artificial Intelligence in Ophthalmology. Springer International Publishing; 2021:177-85.

[32] Zhang XX , Kong J, Yun K. Prevalence of diabetic nephropathy among patients with type 2 diabetes mellitus in China: a meta-analysis of observational studies. J Diabetes Res. 2020;2020:2315607. 10.1155/2020/2315607 32090116 PMC7023800

[33] Wang T , Xi Y, Lubwama R, Hannanchi H, Iglay K, Koro C. Chronic kidney disease among US adults with type 2 diabetes and cardiovascular diseases: a national estimate of prevalence by KDIGO 2012 classification. Diabetes Metab Syndr. 2019;13(1):612-615. 10.1016/j.dsx.2018.11.026 30641775

[34] Liu YT , Hung TY, Lee YK, Huang MY, Hsu CY, Su YC. Association between chronic kidney disease and risk of cataract: a nationwide retrospective cohort study. Am J Nephrol. 2017;45(6):524-531. 10.1159/000475555 28528337

[35] Kang EY , Hsieh YT, Li CH, et al Deep learning-based detection of early renal function impairment using retinal fundus images: model development and validation. JMIR Med Inform. 2020;8(11):e23472. 10.2196/23472 33139242 PMC7728538

[36] Kuo CC , Chang CM, Liu KT, et al Automation of the kidney function prediction and classification through ultrasound-based kidney imaging using deep learning. NPJ Digit Med. 2019;2:29. 10.1038/s41746-019-0104-2 31304376 PMC6550224

