# Correction to: Deep learning algorithms to detect diabetic kidney disease from retinal photographs in multiethnic populations with diabetes

**DOI:** 10.1093/jamia/ocae012

**Published:** 2024-01-24

This is a correction to: Bjorn Kaijun Betzler, Evelyn Yi Lyn Chee, Feng He, Cynthia Ciwei Lim, Jinyi Ho, Haslina Hamzah, Ngiap Chuan Tan, Gerald Liew, Gareth J McKay, Ruth E Hogg, Ian S Young, Ching-Yu Cheng, Su Chi Lim, Aaron Y Lee, Tien Yin Wong, Mong Li Lee, Wynne Hsu, Gavin Siew Wei Tan, Charumathi Sabanayagam, Deep learning algorithms to detect diabetic kidney disease from retinal photographs in multiethnic populations with diabetes, Journal of the American Medical Informatics Association, Volume 30, Issue 12, December 2023, Pages 1904–1914, https://doi.org/10.1093/jamia/ocad179.

In the originally published version of this manuscript, there were errors in the funding statement, which read: This study was supported by the National Medical Research Council (NMRC/STaR/003/2008, NMRC/OFLCG/001/ 2017, and NMRC/MOH-HCSAINV21jun-0001) and the National Research Foundation, Singapore under its AI Singapore Programme (AISG Award No: AISG-GC-2019-001-2A).

This has been corrected to: This study was supported by the Singapore Ministry of Health’s National Medical Research Council (NMRC/STaR/003/2008, NMRC/OFLCG/MOH-001327-03, and NMRC/MOH-HCSAINV21jun-0001) and the National Research Foundation, Singapore under its AI Singapore Programme (AISG Award No: AISG-GC-2019-001-2A).

