# Supplementary material for: Deep learning algorithms to detect diabetic kidney disease from retinal photographs in multiethnic populations with diabetes
Source: J Am Med Inform Assoc. 2023 Sep 2;30(12):1904–14. doi: 10.1093/jamia/ocad179 (PMC10654858; doi:10.1093/jamia/ocad179)
Supplement: ocad179_Supplementary_Data [file ocad179_supplementary_data.docx]

**SUPPLEMENTARY MATERIALS**

| **Supplementary Table S1. Classification performance of the three models by CKD stages at 80% sensitivity or 80% specificity** | | | | | | | |
| --- | --- | --- | --- | --- | --- | --- | --- |
|  | **Sample size, N** | **Accuracy** **when sensitivity = 0.80** | | | **Accuracy when specificity = 0.80** | | |
|  |  | Image-only | RF-only | Hybrid | Image-only | RF-only | Hybrid |
| SiDRP |  |  |  |  |  |  |  |
| - No CKD | 7928 | 0.70 | 0.75 | 0.77 | 0.80 | 0.80 | 0.80 |
| - Stage G3a | 2973 | 0.76 | 0.76 | 0.76 | 0.66 | 0.71 | 0.73 |
| - Stage G3b | 1768 | 0.82 | 0.82 | 0.82 | 0.73 | 0.77 | 0.79 |
| - Stage G4 | 558 | 0.88 | 0.90 | 0.89 | 0.82 | 0.86 | 0.87 |
| - Stage G5 | 57 | 0.82 | 0.78 | 0.80 | 0.77 | 0.73 | 0.78 |
| SEED |  |  |  |  |  |  |  |
| - No CKD | 1171 | 0.57 | 0.65 | 0.69 | 0.80 | 0.80 | 0.80 |
| - Stage G3a | 461 | 0.76 | 0.78 | 0.77 | 0.55 | 0.60 | 0.64 |
| - Stage G3b | 233 | 0.82 | 0.82 | 0.81 | 0.59 | 0.69 | 0.71 |
| - Stage G4 | 79 | 0.87 | 0.82 | 0.86 | 0.73 | 0.73 | 0.79 |
| - Stage G5 | 25 | 0.96 | 0.73 | 0.82 | 0.72 | 0.59 | 0.82 |
| SMART2D |  |  |  |  |  |  |  |
| - No CKD | 485 | 0.51 | 0.51 | 0.59 | 0.80 | 0.80 | 0.80 |
| - Stage G3a | 67 | 0.64 | 0.87 | 0.75 | 0.40 | 0.46 | 0.49 |
| - Stage G3b | 57 | 0.81 | 0.67 | 0.70 | 0.51 | 0.47 | 0.51 |
| - Stage G4 | 44 | 0.93 | 0.86 | 0.91 | 0.64 | 0.39 | 0.73 |
| - Stage G5 | 59 | 0.86 | 0.80 | 0.86 | 0.63 | 0.27 | 0.59 |
| SiDRP = Singapore Integrated Diabetic Retinopathy Program. SEED = Singapore Epidemiology of Eye Diseases. SMART2D = Singapore Macroangiopathy and Microvascular Reactivity in Type 2 Diabetes data.  RF-only: Logistic regression adjusted for age, sex, ethnicity, diabetes duration, HbA1c, and systolic blood pressure.  Hybrid: Logistic regression adjusted for age, sex, ethnicity, diabetes duration, HbA1c, systolic blood pressure, and the predicted z scores from image-only model. | | | | | | | |

| **Supplementary Table S2.** **Characteristics of AHES and NICOLA participants** | | | | | |
| --- | --- | --- | --- | --- | --- |
|  | SiDRP | AHES | p-value | NICOLA | p-value |
| Number of visits | N=13284 | N=460 |  | N=265 |  |
| Number of unique participants | 6066 | 460 |  | 265 |  |
| Number of images | 26568 | 920 |  | 530 |  |
| Case: control | 5356: 7928 | 96:364 |  | 60:205 |  |
| Chronic kidney disease |  |  | <0.001 |  | <0.001 |
| - Stage 3 | 4741 (88.5) | 74 (77.1) |  | 55 (91.7) |  |
| - Stage 4 | 558 (10.4) | 16 (16.7) |  | 4 (6.7) |  |
| - Stage 5 | 57 (1.1) | 6 (6.2) |  | 1 (1.7) |  |
| Age (years) | 64.1 (10.8) | 63.3 (10.9) | 0.127 | 65.9 (8.5) | 0.008 |
| Sex |  |  | <0.001 |  | <0.001 |
| - Female | 6526 (49.1) | 121 (26.3) |  | 90 (34.0) |  |
| - Male | 6758 (50.9) | 339 (73.7) |  | 175 (66.0) |  |
| Duration of Diabetes (years) | 7.0 [3.0, 12.0] | NA | NA | 8.0 [3.8, 15.0] | 0.002 |
| Hemoglobin A1C (%) | 7.2 (1.2) | 7.9 (1.9) | <0.001 | 7.7 (1.7) | <0.001 |
| eGFR (mL/min per 1·73 m2) | 74.1 (27.1) | 78.9 (24.0) | <0.001 | 75.2 (19.9) | 0.499 |
| Systolic blood pressure (mm Hg) | 130.2 (15.9) | 116.1 (17.8) | <0.001 | 136.3 (18.7) | <0.001 |
| Data are n (%), mean (standard deviation, SD), or median [interquartile range, IQR]. P-values were calculated using Pearson’s Chi-squared Test or Student’s t-Test as appropriate for the variable. SiDRP = Singapore Integrated Diabetic Retinopathy Program. | | | | | |

**Supplementary Figure S1. ROC curves for prediction of AHES and NICOLA diabetic kidney disease in image-only, RF-only, and hybrid models.**

**
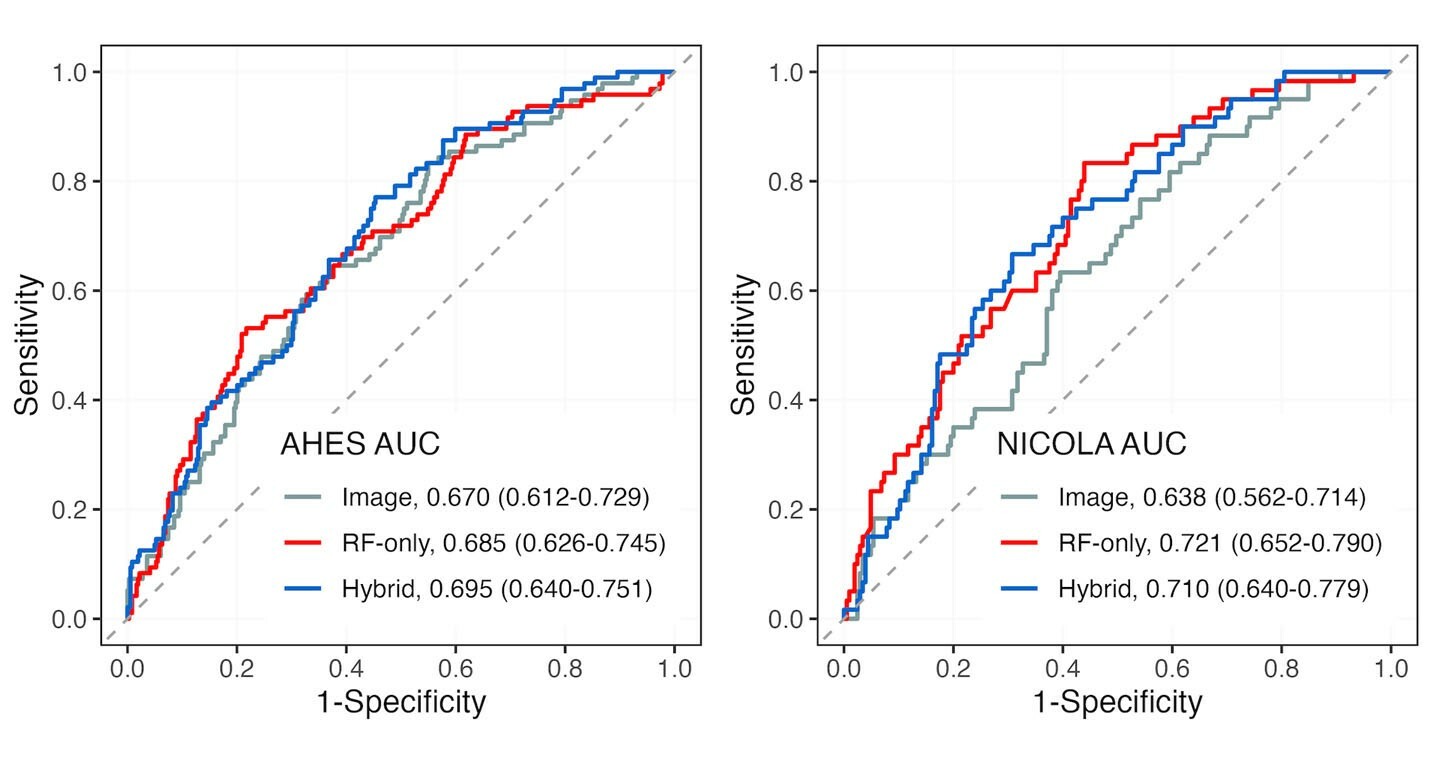
**
